# From sequences to species: Charting the phytoplasma classification and taxonomy in the era of taxogenomics

**DOI:** 10.3389/fmicb.2023.1123783

**Published:** 2023-03-09

**Authors:** Kiran Kirdat, Bhavesh Tiwarekar, Shivaji Sathe, Amit Yadav

**Affiliations:** ^1^National Centre for Cell Science, NCCS Complex, Savitribai Phule Pune University, Pune, India; ^2^Department of Microbiology, Tuljaram Chaturchand College, Baramati, India

**Keywords:** phytoplasma taxonomy, 16S rRNA gene, MLST, OGRI, genome phylogeny, the uncultivated Code

## Abstract

Phytoplasma taxonomy has been a topic of discussion for the last two and half decades. Since the Japanese scientists discovered the phytoplasma bodies in 1967, the phytoplasma taxonomy was limited to disease symptomology for a long time. The advances in DNA-based markers and sequencing improved phytoplasma classification. In 2004, the International Research Programme on Comparative Mycoplasmology (IRPCM)- Phytoplasma/Spiroplasma Working Team – Phytoplasma taxonomy group provided the description of the provisional genus ‘*Candidatus* Phytoplasma’ with guidelines to describe the new provisional phytoplasma species. The unintentional consequences of these guidelines led to the description of many phytoplasma species where species characterization was restricted to a partial sequence of the 16S rRNA gene alone. Additionally, the lack of a complete set of housekeeping gene sequences or genome sequences, as well as the heterogeneity among closely related phytoplasmas limited the development of a comprehensive Multi-Locus Sequence Typing (MLST) system. To address these issues, researchers tried deducing the definition of phytoplasma species using phytoplasmas genome sequences and the average nucleotide identity (ANI). In another attempts, a new phytoplasma species were described based on the Overall Genome relatedness Values (OGRI) values fetched from the genome sequences. These studies align with the attempts to standardize the classification and nomenclature of ‘*Candidatus’* bacteria. With a brief historical account of phytoplasma taxonomy and recent developments, this review highlights the current issues and provides recommendations for a comprehensive system for phytoplasma taxonomy until phytoplasma retains ‘*Candidatus’* status.

## Introduction

Phytoplasmas (Kingdom, Bacteria; Phylum, Mycoplasmatota; class, Mollicutes; genus, ‘*Candidatus* Phytoplasma’) are phloem-inhabiting obligate plant pathogens. These are cell wall-less bacterial pathogens transmitted by phloem-feeding insect vectors mainly belonging to *Cicadellidae, Derbidae*, and *Cixiidae*; commonly known as leafhoppers, planthoppers, and psyllids (Weintraub and Beanland, 2006). They can also be transferred by grafting infected plant material or parasitic plants such as dodder (*Cuscuta* sp.) or spread through infected vegetative material used for plant propagation (Marcone et al., 1997; Přibylová and Špak, 2013) or through seeds in some cases (Calari et al., 2011; Kirdat et al., 2022). The infected plants show symptoms like brooming (excessive branching or tillering), virescence (abnormal development of green pigmentation in floral parts of a plant), little leaf (reduction in leaf size), phyllody (abnormal growth of floral parts into vegetative structures) and chlorosis resulting in yellow coloration of leaves (Lee et al., 2000; Rao et al., 2017). Phytoplasma causes diseases over thousands of plant species worldwide, many of which are lethal. In the agriculture and horticulture sectors globally, phytoplasma-related diseases lead to extensive yield losses. Many economically important crops, including vegetables, spices, medicinal plants, ornamentals, cash crops, palms, fruit trees, weeds, timber, and shade trees, are affected due to phytoplasma-related diseases (Lee et al., 2000; Rao et al., 2017, 2018; Duduk et al., 2018; Bertaccini, 2022; Brooks et al., 2022; Sundararaj et al., 2022; Marcone et al., 2023).

Phytoplasma diseases have been reported from more than 100 countries, as evident from the sequences available in the GenBank database. The impact of phytoplasma diseases and their distribution in different geographical areas depends on the host range of the phytoplasma and the polyphagous feeding behavior of the insect vector (Weintraub and Beanland, 2006; Foissac and Wilson, 2009). Phytoplasma research has flourished in the last two decades, and reviews published from time to time give a good understanding of phytoplasma studies, including its taxonomy, etiology, transmission, and interaction with insect and plant hosts (Lee et al., 2000; Bai et al., 2006; Weintraub and Beanland, 2006; Hogenhout et al., 2008; Bertaccini and Duduk, 2009; Sugio et al., 2011; Rao et al., 2017; Duduk et al., 2018; Bertaccini et al., 2022; Wei and Zhao, 2022). This review summarizes the history and current status of phytoplasma taxonomy, focusing on the different classification systems used, their advantages and drawbacks; issues related to published phytoplasma species; recent attempts to develop a classification system for *‘Candidatus’* bacteria and related issues in context with phytoplasma.

## Pioneering phytoplasma taxonomy: The early years

The first phytoplasma disease was scientifically recorded 200 years ago when the mulberry dwarf disease outbreak was observed in Japan (Ishiie, 1965; Okuda, 1972). However, the historical record for phytoplasma infection is about 1,000 years old from China, where tree peonies (*Paeonia suffruticosa*) were appreciated as the most attractive flower for a long time (Maramorosch, 2011). During the Song dynasty (960-1,227), special tributes were being paid to the imperial court by offering a variety of green flowers (tree peonies exhibiting floral virescence) as a most precious variety of the plant (Wang and Maramorosch, 1988). Earlier in 1897, a Japanese national research committee struggled to determine the cause of the Mulberry dwarf disease. Since then, many diseases like rice yellow dwarf disease and paulownia witches’ broom disease have been recorded without conclusive identification of the causative agent of these diseases, which were thought to be some undiscovered viruses (Kunkel, 1926; Okuda, 1972; Lee et al., 2000; Maramorosch, 2011). In 1967, a Japanese scientist Doi Y. and his co-workers located pleomorphic bodies in the phloem tissue of plants showing disease symptoms. They named these entities ‘Mycoplasma-like-Organisms’ (MLOs) due to their resemblance with animal mycoplasmas. The causative agent of these diseases was shown to be transmitted by phloem-feeding insects and by grafting (Doi et al., 1967). Further, its sensitivity to tetracycline supported the hypothesis of the existence of MLO and not viruses (Doi et al., 1967; Ishiie et al., 1967). Later, many subsequent studies established that MLOs are associated with diseases in several hundred plant species showing typical symptoms. Researchers correlated these ‘yellows’ diseases associating organisms based on symptomatology and host range. This led to the naming of new MLOs based on the original plant host and symptoms, e.g., Aster Yellows, Apple Proliferation, Elm Yellows, and others.

MLOs resisted the attempts to cultivate them for years and has yet to be successful. Failure to cultivate these organisms drove the developments in phytoplasma taxonomy toward obtaining the marker data using serological and DNA-based techniques. Around 1990, the acquisition of knowledge in DNA biology facilitated the direct detection of MLOs DNA by various techniques such as DNA–DNA hybridization (Kirkpatrick et al., 1987; Razin et al., 1987; Bonnet et al., 1990), Polymerase Chain Reaction (PCR), cloning and sequencing of MLOs DNA fragments (Lee, 1988). A method for PCR amplification of 16S rRNA gene with universal primers was then developed and soon became a routine practice for the detection, identification, diversity, and taxonomy of MLOs (Deng and Hiruki, 1991; Schneider et al., 1995; Gundersen and Lee, 1996; Smart et al., 1996; Lee et al., 1998). These findings eventually simplified the diagnosis and classification of MLOs, leading to a flurry of publications in the 1990s and later.

The MLOs were placed in class Mollicutes (Phylum *Tenericutes*; now *Mycoplasmatota*) due to the lack of a cell wall, small genome size, and low G+C content. In addition, unlike sterol-requiring mycoplasmas, MLOs membranes were shown to resemble membranes of non-sterol-requiring *Acholeplasma* strains. MLOs showed resistance to digitonin and sensitivity toward hypotonic salt solutions (Lim and Sears, 1992). The first gene sequences analyzed also revealed that contrary to mycoplasmas and spiroplasmas, MLOs use UGA as a stop codon, as *Acholeplasma* strains do (Lim and Sears, 1991). It became essential to determine if these MLOs are monophyletic or spread across the class *Mollicutes*. Several studies based on the phylogenetic analysis of 16S rRNA and ribosomal protein (*rp*) gene sequences revealed the phylogenetic relationships of MLOs within the group and with related organisms (Lim and Sears, 1989, 1992; Kirkpatrick et al., 1990; Kuske and Kirkpatrick, 1992; Schneider et al., 1993; Gundersen et al., 1994; Seemuller et al., 1994; Lee et al., 1998, 2003; Seemüller et al., 1998). These studies established that MLOs were evolutionarily distinct from animal mycoplasmas. They form a large discrete monophyletic clade, paraphyletic to the *Acholeplasma* species within the *Anaeroplasma* order.

In 1992, the International Committee on Systematic Bacteriology (ICSB) Subcommittee on the Taxonomy of Mollicutes adopted the trivial name ‘Phytoplasma’ (Tully, 1993). In line with these studies, Gundersen et al. (1994) proposed that MLOs should be represented taxonomically at the minimal genus level. They recognized five major phylogenetic groups with 11 distinct subclades (monophyletic groups or taxa). Those were, Maryland Aster Yellows AY1; Apple Proliferation AP-A; Peanut Witches’-broom PnWB; Canada Peach X CX; Rice Yellow Dwarf RYD; Pigeon Pea Witches’-Broom PPWB; Palm Lethal Yellowing LY; Ash Yellows AshY; Clover Proliferation CP; Elm Yellows EY; and Loofah Witches’-Broom LfWB (Gundersen et al., 1994). Until then, phytoplasmas were named based only on their hostname and the disease symptoms.

## The 16S rRNA gene: The ultimate phytoplasma classifier

A taxonomic note documenting the characteristics of putative prokaryote taxa and implementation of *‘Candidatus’* status was released in 1995 by the International Code of Nomenclature of Bacteria (ICNB). This recommendation allowed the researcher to publish bacterial taxa that cannot be described per norms laid by the ICNB (known as *‘Candidatus’* taxa). The record of the existence of such species was possible only through the detection of DNA sequences obtained from the environmental sample. The taxonomic note suggested recording the structural, metabolic, and reproductive features and the properties of the ecological niche and genomic information used to determine the organism’s phylogenetic position (Murray and Schleifer, 1994; Murray and Stackebrandt, 1995). Following this, several distinct phytoplasma taxa were described, viz. ‘*Candidatus* (*Ca*.) Phytoplasma (P.) aurantifolia’ (Zreik et al., 1995), ‘*Ca.* P. australiense’ (Davis et al., 1997), ‘*Ca.* P. australasia’ (White et al., 1998), ‘*Ca.* P. fraxini’ (Griffiths et al., 1999), ‘*Ca.* P. japonicum’ (Sawayanagi et al., 1999), ‘*Ca.* P. brasiliense’ (Montano et al., 2001), ‘*Ca.* P. castaneae’ (Jung et al., 2002), ‘*Ca.* P. phoenicium’ (Verdin et al., 2003), ‘*Ca.* P. ziziphi’ (Jung et al., 2003a), ‘*Ca.* P. oryzae’ (Jung et al., 2003b) and ‘*Ca.* P. ulmi’ (Lee et al., 2004b). The basis of classification of these species was majorly 16S rRNA gene sequence similarity, RFLP pattern of 16S rRNA gene, and in some cases, analysis of 16S-23S spacer region, serology, and biological properties (Table 1). In 2004, the International Research Programme on Comparative Mycoplasmology (IRPCM)- Phytoplasma/Spiroplasma Working Team- Phytoplasma taxonomy group provided the description of the provisional genus ‘*Ca.* Phytoplasma’ based on the near-full length sequence of phytoplasma 16S rRNA gene (Firrao, 2004). As per IRPCM recommendations, a novel ‘*Ca.* Phytoplasma’ species description should refer to a single, unique 16S rRNA gene sequence of >1,200 bp and share <97.5% sequence similarity to any previously described ‘*Ca.* Phytoplasma’ species unless the phytoplasma under consideration represents an ecologically distinct population (Firrao, 2004). Later, several phytoplasma species were described, primarily based on the 16S rRNA gene phylogeny and its RFLP-based classification (Table 1).

**TABLE 1:** List of provisional *‘Candidatus* Phytoplasma’ species published as of December 2022.

Provisional species name	Reference strain (Accession number)	Reference strain gene sequences (Accession number)	Name of the disease (Plant host)	Insect vector(s)	References
‘*Ca*. Phytoplasma citri’ formerly, ‘*Ca*. Phytoplasma aurantifolia’	WBDL (U15442)	404 proteins NZ_MWKN00000000.1	Lime Witches’-broom (*Citrus aurantifolia*)	Not reported	Zreik et al., 1995; Oren, 2017
‘*Ca.* Phytoplasma australiense’	AUSGY (L76865)	Not reported	Australian Grapevine Yellows (*Vitis vinifera* L.)	Not reported	Davis et al., 1997
‘*Ca.* P. australasiaticum’ formerly ‘*Ca.* Phytoplasma australasia’	PpYC (Y10097)	Not reported	Papaya Yellow Crinkle (*Carica papaya*)	Not reported	White et al., 1998
‘*Ca.* Phytoplasma fraxini’	AshY1 (AF092209)	Not reported	Ash Yellows (*Fraxinus* sp.)	Not reported	Griffiths et al., 1999
‘*Ca.* Phytoplasma japonicum’	JHP (AB010425)	Not reported	Japanese hortensia Phyllody (*Hydrangea* sp.)	Not reported	Sawayanagi et al., 1999
‘*Ca.* Phytoplasma brasiliense’	HibWB26 (AF147708)	Not reported	Hibiscus witches’ broom (*Hibiscus rosa-sinensis*)	Not reported	Montano et al., 2001
‘*Ca.* Phytoplasma castaneae’	CnWB (AB054986)	Not reported	Chestnut witches’ broom (*Castanea crenata*)	Not reported	Jung et al., 2002
‘*Ca.* Phytoplasma oryzae’	RYD-J (D12581)	Not reported	Rice Yellow Dwarf (*Oryza sativa*)	*Nephotettix cincticeps*, *N*. *virescens* and *N*. *nigropictus*	Jung et al., 2003b
‘*Ca.* Phytoplasma ziziphi’	JWB-G1 (AB052876)	Not reported	Jujube Witches’-Broom (*Zizyphus jujuba*)	*Hishimonus sellatus*	Jung et al., 2003a
‘*Ca.* Phytoplasma phoenicium’	A4 (AF515636)	Not reported	Lethal of Almond Trees (*Prunus amygdalus*)	Not reported	Verdin et al., 2003
‘*Ca.* Phytoplasma trifolii’	CP(AY390261)	Not reported	Clover Proliferation (*Trifolium hybridum*)	*Macrosteles fascifrons*	Hiruki and Wang, 2004
‘*Ca.* Phytoplasma asteris’	OAY (M30790)	*rpl23*, *rpl2*, *rps19*, *rpl22*, *rps3*, *rpl16* (M74770)	Aster Yellows (*Catharanthus roseus*)	*Macrosteles quadrilineatus*, *Euscelis* sp., *Scaphytopius* sp. and *Aphrodes* sp.	Lee et al., 2004a
‘Ca. Phytoplasma ulmi’	EY1 (AY197655)	*rpl22*–*rps3* (AY197675) *secY* (AY197690)	Elm Yellows (*Ulmus* sp.)	*Scaphoideus luteolus*	Lee et al., 2004b
‘*Ca.* Phytoplasma cynodontis’	BGWL-C1 (AJ550984) LW01 (LT558776)	425 proteins of LW01 (VWOH00000000)	Bermuda Grass White Leaf (*Cynodon dictylon* L.)	Not reported	Marcone et al., 2004b; Kirdat et al., 2021
‘*Ca.* Phytoplasma spartii’	SpaWB (X92869)	Not reported	Spartium Witches’-broom (*Spartium junceum*)	Not reported	Marcone et al., 2004a
‘*Ca.* Phytoplasma rhamni’	BWB (AJ583009)	Not reported	Buckthorn witches’-broom (*Rhamnus catharticus*)	Not reported	Marcone et al., 2004a
‘*Ca.* Phytoplasma allocasuarinae’	AlloY (AY135523)	Not reported	Allocasuarina yellows Witches’-broom (*Allocasuarina muelleriana*)	Not reported	Marcone et al., 2004a
‘*Ca.* Phytoplasma mali’	AP15 (AJ542541)	Not reported	0Apple proliferation (*Malus domestica*)	*Cacopsylla picta* and *Cacopsylla melanoneura*	Seemüller and Schneider, 2004
‘*Ca.* Phytoplasma pyri’	PD1 (AJ542543)	Not reported	Pear Decline (*Pyrus communis*)	*Cacopsylla pyricola* and *Cacopsylla pyri*	Seemüller and Schneider, 2004
‘*Ca.* Phytoplasma prunorum’	ESFY-G1 (AJ542544)	Not reported	European Stone Fruit Yellows (*Prunus persica*)	*Cacopsylla pruni*	Seemüller and Schneider, 2004
‘*Ca.* Phytoplasma graminis’	SCYLP (AY725228)	Not reported	Sugarcane yellow leaf syndrome (*Saccharum officinarum*)	*Saccharosydne saccharivora*	Pin et al., 2005
‘*Ca.* Phytoplasma caricae’	PAY (AY725234)	Not reported	Papaya bunchy top (*Carica papaya*)	*Empoasca papayae* Oman	Pin et al., 2005
‘*Ca.* Phytoplasma pini’	Pin127S (AJ632155)	Not reported	Phytoplasma infection of pine tree (*Pinus halepensis*)	Not reported	Schneider and Torres, 2005
‘*Ca.* Phytoplasma americanum’	APPTW12-NE (DQ174122)	Not reported	Potato purple top wilt (*Solanum tuberosum*)	Not reported	Lee et al., 2006a
‘*Ca.* Phytoplasma fragariae’	StrawY (DQ086423)	Not reported	Yellows diseased strawberry (Fragaria*ananassa)	Not reported	Valiunas et al., 2006
‘*Ca.* Phytoplasma lycopersici’	THP (EF199549)	Not reported	Parsley Leaf of Tomato (*Lycopersicon esculentum* L.)	Not reported	Arocha et al., 2007
‘*Ca.* Phytoplasma omanense’	IM-1 (EF666051)	Not reported	Cassia witches’-broom (*Cassia italica*)	Not reported	Al-saady et al., 2008
‘*Ca.* Phytoplasma tamaricis’	SCWB1 (FJ432664)	*tilS* (FJ432664)	Witches’-broom-disease (*Tamarix chinensis* Lour.)	Not reported	Zhao et al., 2009a
‘*Ca.* Phytoplasma costaricanum’	SoyST1c1 (HQ225630)	Not reported	Soybean stunt (*Glycine max*)	Not reported	Lee et al., 2011
‘*Ca.* Phytoplasma rubi’	RuS (AY197648)	*Tuf*, *rpl22*, *rps3*, *rps8*, *rpl6*, *rpl18*, *secY*, *map*, *uvrB*, *degV* (KR233474 to 78)	Rubus stunt (*Rosa canina*)	Not reported	Malembic-Maher et al., 2011
‘*Ca.* P. australamericanum’ formerly ‘Ca. Phytoplasma sudamericanum’	PassWB-Br3 (GU292081)	Not reported	Passion fruit witches’-broom (*Passiflora edulis* f. flavicarpa Deg)	Not reported	Davis et al., 2012
‘*Ca.* Phytoplasma convolvuli’	BY-S57/11 (JN833705)	Not reported	Bindweed yellows (*Convolvulus arvensis*)	Not reported	Maixner et al., 2012
‘*Ca.* Phytoplasma pruni’	PX11CT1 (JQ044393) rrnA, (JQ044392) rrnB	*secY* (JQ268254), *rp* (JQ360960)	X-disease of stone fruits (*Prunus persica*)	Not reported	Davis et al., 2013
‘*Ca.* Phytoplasma malaysianum’	MaPV (EU371934)	Not reported	Virescence and phyllody of Madagascar periwinkle (*Catharanthus roseus*)	Not reported	Nejat et al., 2013
‘*Ca.* Phytoplasma solani’	STOL11 (AF248959)	*tuf* (JQ797670), *secY* (JQ797668), *rp* (JQ797662)	Stolbur (*Capsicum annuum*)	*Hyalesthes obsoletus*, *Pentas tiridiusbeieri* and *Macrosteles quadripunctulatus*	Quaglino et al., 2013
‘*Ca.* Phytoplasma balanitae’	BltWB (AB689678)	*rp* (AB689679), *secY* (AB689680)	Balanites witches’ broom (*Balanites triflora*)	Not reported	Win et al., 2013
‘*Ca.* Phytoplasma palmicola’	LYDM-178 (KF751387)	Not reported	lethal yellowing-type disease of coconut (*Cocos nucifera* L.)	Not reported	Harrison et al., 2014
‘*Ca.* Phytoplasma hispanicum’	MPV (AF248960)	Not reported	Periwinkle virescence (*Catharanthus roseus*)	Not reported	Davis et al., 2016
‘*Ca.* Phytoplasma meliae’	ChTY-Mo3 (KU850940)	*secA* (KU850944), *rpLV-rpsC* (KU850948)	Chinaberry yellowing (*Melia azedarach* L.)	Not reported	Fernández et al., 2016
‘*Ca.* Phytoplasma cirsii’	CirYS (KR869146)	partial *secA* (KU557489)	Yellowing, stunting, inflorescence and proliferation (*Cirsium arvense* (L.) Scop.) and virescence, phyllody, deformations (*Dahlia* sp.)	Not reported	Šafářová et al., 2016
‘*Ca.* Phytoplasma wodyetiae’	Bangi-2 (KC844879)	Not reported	Yellow decline disease (*Wodyetia bifurcata*)	Not reported	Naderali et al., 2017
‘*Ca.* Phytoplasma luffae’	LfWB (AF248956) rrnA, (AF353090) rrnB	Not reported	loofah witches’ broom (*Luffa aegyptica* Mill)	Not reported	Davis et al., 2017
‘*Ca.* Phytoplasma noviguineense’	BCS-Bo (LC228755)	*secY* (LC228769), *rp* (LC228762)	Bogia coconut syndrome (*Cocos nucifera*) and banana wilt disease (*Musa* sp.)	Species from the families *Delphacidae, Derbidae, Flatidae, Lophopidae, Pentatomidae*, and *Ricaniidae*	Miyazaki et al., 2018
‘*Ca.* Phytoplasma stylosanthis’	VPRI 43683 (MT431550)	*tuf* (MT432813), *secA* (MT432821), *rps19-rpl22-rps3* (MT461153)	Little leaf (*Solanum tuberosum* L.)	Not reported	Rodrigues Jardim et al., 2020
‘*Ca.* Phytoplasma sacchari’	SCGS (MN889545)	404 proteins (VWXM00000000)	Sugarcane Grassy Shoot (*Saccharum officinarum* L.)	*Matsumura tettixhiroglyphicus*, *Yamatotettix flavovittatus* and *Maiestasportica*	Kirdat et al., 2021
‘*Ca.* Phytoplasma tritici’	WBD (AVAO01000003)	500 proteins (NZ_AVAO00000000)	Wheat blue dwarf (*Triticum aestivum* L.)	*Psammotettix striatus*	Zhao et al., 2021
‘*Ca.* Phytoplasma dypsidis’	RID7692 (MT536195)	*rps19*, *rpl22*, *rps3* (MT304824)	lethal wilt disease (*Dypsis poivreana*)	Not reported	Jones et al., 2021

The list does not contain species names which were not formally described; they are ‘*Ca*. Phytoplasma cocosnigeriae’ (LDN, Y14175), ‘*Ca*. Phytoplasma cocostanzaniae’ (LD, X80117), ‘*Ca*. Phytoplasma palmae’ (Coconut lethal yellowing MLO, U18747) and ‘*Ca*. Phytoplasma vitis’ (FD70, AF176319). The 16S rRNA gene of strain LDN (Y14175) of proposed provisional species, ‘*Ca*. Phytoplasma cocosnigeriae’ matches 100% with ‘*Ca*. P. palmicola’ (LYDM-178, KF751387).

Meanwhile, a ‘revised’ classification of phytoplasmas was founded on RFLP analyses of 16S rRNA and ribosomal protein gene sequences (Lee et al., 1998). They differentiated phytoplasma strains into 14 major ‘16Sr groups’ based on RFLP profiles generated using 17 restriction enzymes. This approach provided a simple and rapid method for differentiation and classification of phytoplasmas compared to laborious methods involving DNA probes, DNA sequencing, and serological methods affected with inefficacy. Thus, the classification based on 16S rRNA gene phylogeny and its RFLP analysis was widely accepted by researchers. Further, the computer-simulated RFLP analysis was devised, including *in silico* restriction digestion of obtained 16S rRNA gene sequence, virtual gel plotting, and a similarity coefficient based on the virtual RFLP pattern (Wei et al., 2007). Soon an interactive online tool, *iPhyClassifier*, was introduced to ramp up the speed and capacity of the 16S rRNA gene sequence-based phytoplasma classification system (Wei et al., 2007, 2008; Zhao et al., 2009b).

This RFLP-based phytoplasma classification system considers mutations at restriction sites where an uneven weightage of nucleotide positions ignores the information available in a full-length sequence, and internal variation in a sequence is not deemed. If there is a single nucleotide change in the sequence at the restriction site, there are chances that the RFLP system classifies that as a new subgroup, even if the overall sequence similarity is 99% or more. Additionally, it is well known that many phytoplasmas have two rRNA operons (termed as rrnA and rrnB), and interoperon sequence heterogeneity exists in some strains (Seemuller et al., 1994; Firrao and Smart, 1996; Liefting et al., 1996; Davis and Sinclair, 1998; Oshima et al., 2004; Bai et al., 2006). Sequence variations between heterogeneous rrnA operons may assign the same phytoplasma strain to different RFLP subgroups. For example, the phytoplasma strain DY2014 contains two heterogeneous 16S rRNA genes. The *iPhyClassifier* classifies them into two sub-groups, although they differ by only one base pair and belong to the same phytoplasma strain (Cho et al., 2019). To avoid confusion, a three-letter subgroup designation system was proposed for such stains (Wei et al., 2008). However, confusion persisted on what criteria should be used to decide the formal subgroup of a strain.

The formation of group and subgroup is based on the cut-off values of RFLP similarity coefficients (SC) (Lee et al., 1998). The RFLP-SC compares the restriction patterns of 16S rRNA gene sequences with the reference sequence and assigns it to the existing group/subgroup or suggests forming a new group/subgroup. A new phytoplasma 16Sr group and subgroup can be created if the RFLP coefficient is less than 0.85 and 0.97, respectively (Lee et al., 1998; Wei et al., 2007; Zhao et al., 2009b). However, confusion remains with the validity of some phytoplasma groups/subgroups as the basis of their formation remains unclear. Many ‘Aster Yellow’ phytoplasma strains have RFLP-SC ranging from 0.85 to 0.97 and were designated as 16SrXII subgroup strains (for example, but not limited to AF222065, HM067754, AP006628, JQ730859, MT106667, AF222066, AY265213, AF503568, HM067755, and CP000061). At the same time, they correspond to subgroups strains related to group 16SrI. Similar is the case with phytoplasma strains related to the 16SrV and 16SrVI groups. In another case, the reference strains of 16SrXIV-A group, strain BGWL-C1 (AJ550984), show RFLP-SC of 0.87 compared with reference sequences of subgroup 16SrXI-A (AB052873), indicating being 16SrXI group member. Therefore, since its formation in 1998, no subgroup strain could be added to the 16SrXIV group based on RFLP-SC-based classification. In 2016, ICSB-Mollicutes acknowledged the shortcomings of the taxonomic justification for phytoplasma nomenclature based on RFLP ‘barcoding’ (May et al., 2017).

## The orphan phytoplasma species, the unintentional consequences

**FIGURE 1:**
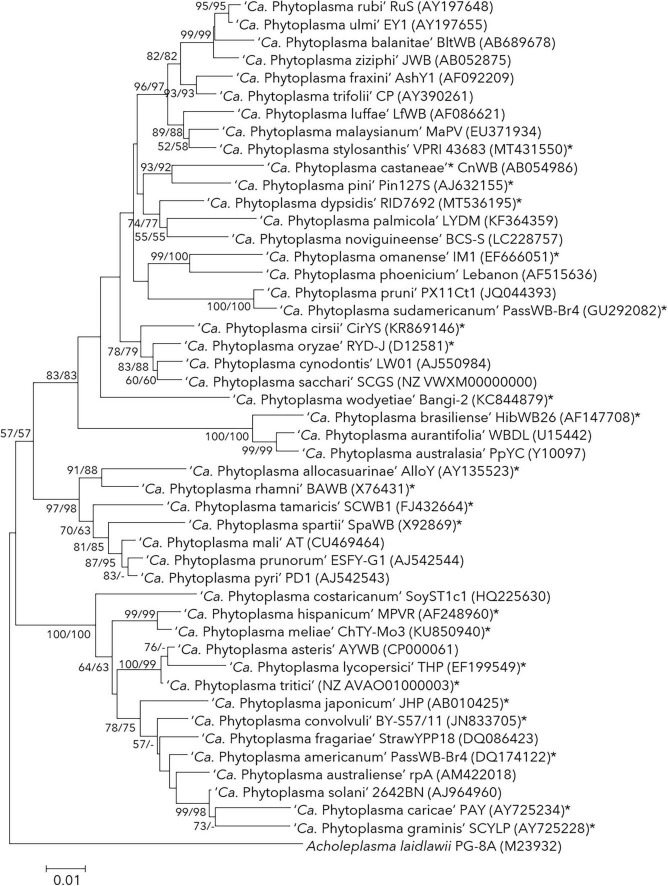
Phylogenetic tree inferred from analysis of reference 16S rRNA gene sequences of published phytoplasma species. The neighbour-joining (NJ) and maximum-likelihood (ML) methods were used to build the phylogenetic tree building using MEGA 7 (Kumar et al., 2016). The topologies of the trees were evaluated by bootstrap analysis based on 1000 replicates. Figures at nodes (>50) of the branches indicate the percentage of replicate trees obtained from NJ and ML methods. The absence of a branch at a particular node was marked by ‘- ’. There was a total of 976 positions in the final dataset. The 16S rRNA sequence of *Acholeplasma laidlawii* PG-8A (M23932) was used as an outgroup. Bar indicates the number of nucleotide substitutions per site. *Indicates orphan species described in text.

The International Code of Nomenclature of Prokaryotes (ICNP) required every taxonomic rank to be associated with type strains and type material (detailed description, illustration or non-viable specimen) in some cases. The deposition of viable pure cultures into two international culture collections as type material was made mandatory. The unintended consequences of this amendment led to denial of use of code for many endosymbionts which were not in the form of axenic culture. The type strain (in the case of cultivable bacteria) or reference strain (in ‘*Candidatus* bacteria’) is a formal representative of organisms at the species rank. A bacterial species form a ‘monophyletic cluster’ containing a group of strains that share a most recent common ancestor with common phenotypic and genomic characteristics (Rosselló-Mora and Amann, 2001; Torsvik et al., 2002). Therefore, it is vital to consider the population dynamics (diversity, abundance, and endemism) of the isolates while describing the new taxon through reference strain description. The type strain alone cannot and should not represent the species clade representing the non-existing intra-species diversity. Among phytoplasma species published, most were described based on the 16S rRNA gene phylogeny (Table 1 and Figure 1). Many reference 16S rRNA gene sequences of reference phytoplasma strains matched to no other 16S rRNA sequences of ‘-related’ strains reported earlier or later of the publication from the same or another geographic location, making them orphan species. They are, ‘*Ca.* P. japonicum’ (Sawayanagi et al., 1999), ‘*Ca.* P. brasiliense’ (Montano et al., 2001), ‘*Ca.* P. castaneae’ (Jung et al., 2002), ‘*Ca.* P. oryzae’ (Jung et al., 2003b), ‘*Ca.* P. spartii,’ ‘*Ca.* P. rhamni,’ ‘*Ca.* P. allocasuarinae’ (Marcone et al., 2004a), ‘*Ca.* P. graminis’ (Pin et al., 2005), ‘*Ca.* P. caricae’ (Arocha et al., 2005) ‘*Ca.* P. pini’ (Schneider and Torres, 2005), ‘*Ca.*’ P. americanum’ (Lee et al., 2006a), ‘*Ca.* P. lycopersici’ (Arocha et al., 2007), ‘*Ca.* P. omanense’ (Al-saady et al., 2008), ‘*Ca.* P. tamaricis’ (Zhao et al., 2009a), ‘*Ca.* P. sudamericanum’ (Davis et al., 2012), ‘*Ca.* P. convolvuli’ (Maixner et al., 2012), ‘*Ca.* P. hispanicum’ (Davis et al., 2016), ‘*Ca.* P. meliae’ (Fernández et al., 2016), ‘*Ca.* P. cirsii’ (Šafářová et al., 2016), ‘*Ca.* P. wodyetiae’ (Naderali et al., 2017), ‘*Ca.* P. stylosanthis’ (Rodrigues Jardim et al., 2020), ‘*Ca*. P. dypsidis’ (Jones et al., 2021) and ‘*Ca*. P. tritici’ (Zhao et al., 2021) (Table 1).

Phylogenetic analysis has helped phytoplasma taxonomists understand its evolution and find a common ancestor. Most phytoplasma phylogenetic studies are based on DNA sequences, and therefore sequence quality remains vital for accurate phylogenetic analysis. The phylogenetic position of ‘*Ca.* P. graminis,’ ‘*Ca.* P. caricae,’ ‘*Ca.* P. lycopersici’ and ‘*Ca.* P. wodyetiae’ are speculated to be ‘unusual’ with atypical terminal branching from its ancestor and low bootstrap values at the branch node (Figure 1). The description of ‘*Ca.* P. wodyetiae’ was based on the analysis of the 16S rRNA gene and RFLP of its reference strain BANGI-2. This phytoplasma was detected from the foxtail palm (*Wodyetia bifurcata*), showing ‘Foxtail Palm Yellow Decline (FPYD)’ disease, which was infected with two known phytoplasmas, ‘*Ca.* P. asteris’ (Lee et al., 2004a) and ‘*Ca.* P. cynodontis’ (Marcone et al., 2004b). The reference 16S rRNA gene sequence (KC844879) of ‘*Ca.* P. wodyetiae’ is probably a ‘chimeric’ sequence made up of partial 16S rRNA gene sequences from ‘*Ca*. P. asteris’ and ‘*Ca.* P. cynodontis’ when analyzed using chimera detection tools, Mallard (Ashelford et al., 2006) and Pintail (Ashelford et al., 2005) (unpublished data). As mentioned, no other phytoplasma strains were reported earlier or in subsequent studies related to these species, either represented by the 16S rRNA or other gene sequences.

## Multi-locus sequence analysis (MLSA): Phylogenetics beyond 16S rRNA gene

Although the 16S rRNA gene is regarded as a universal genetic marker for prokaryote classification, it faces genetic saturation due to its restricted length compared to genome size. The number of mutable sites is limited due to functional constraints. This issue can be addressed using additional phylogenetic markers, essentially housekeeping genes. In the last decade, several other conserved genes were evaluated in the search for molecular markers for adequate differentiation of various phytoplasmas found across geographic areas and plant hosts (Marcone et al., 2000; Lee et al., 2004a, 2006b; Martini et al., 2007). The description of the first phytoplasma species, ‘*Ca.* P. aurantifolia,’ was based on its 16S rRNA gene sequences and the the16S-23S ribosomal DNA spacer region (Zreik et al., 1995). The hybridization profiles of the reference strain were obtained by using MLO-specific probes, and genome size was derived to describe ‘*Ca.* P. aurantifolia’ and the ‘*Ca.* P. fraxini’ in addition to the 16S rRNA gene analysis (Griffiths et al., 1999). The analysis of the 16S-23S ribosomal DNA spacer region was used additionally in the case of ‘*Ca.* P. trifolii’ (Hiruki and Wang, 2004), ‘*Ca.* P. cynodontis’ (Marcone et al., 2004b), ‘*Ca.* P. mali,’ ‘*Ca.* P. pyri’ and ‘*Ca.* P. prunorum’ (Seemüller and Schneider, 2004). The sequences of the elongation factor (EF) and Tu (*tuf*) genes and ribosomal protein (*rp*) genes were used for the first time for the description of the ‘*Ca.* P. asteris’ in addition to the 16S rRNA gene sequence analysis (Lee et al., 2004a). Later, *secY*, *tuf*, *uvrB* -*degV*, and *rp* gene sequences (either or more) were used to describe ‘*Ca.* P. ulmi’, (Lee et al., 2004b), ‘*Ca.* P. rubi’ (Malembic-Maher et al., 2011), ‘*Ca.* P. solani’ (Quaglino et al., 2013), ‘*Ca.* P. balanitae’ (Win et al., 2013), ‘*Ca.* P. meliae,’ ‘*Ca.* P. cirsii’ and ‘*Ca.* P. stylosanthis’. Average nucleotide identity (ANI) value, *amp*, and *secY* were used to describe ‘*Ca*. P. tritici’ while ‘*Ca*. P. dypsidis’ was described using the 16S rRNA and ribosomal protein genes.

Phylogenetic analysis of housekeeping genes confirmed and strengthened the 16S rRNA gene-based classification systems and improvised finer strain differentiation with more discrete branches. At the same time, none of the studies was evaluated for the performance of the marker genes by considering their variable rates of evolution by comparing other genes present in the phytoplasma genome (including the 16S rRNA gene) used for a multi-locus classification system. Therefore, it was essential to evaluate these marker genes for genome-level assessment for their relative performance in classifying the phytoplasma strains, considering their evolutionary relationships (Martini et al., 2019). It is more likely that any single gene’s evolutionary history may differ from the phylogenetic history of the whole organism as they do not account for the horizontal gene transfer and unrecognized paralogy. Most published markers described for particular groups or species may not necessarily be suitable for another group of phytoplasma species. A five-gene markers MLSA for Aster Yellow (16SrI, AY) group of phytoplasmas to supplement the 16S rRNA gene phylogeny was proposed to improvise the phytoplasma taxonomy (Cho et al., 2020). These proposed marker genes [Replication initiation protein DnaD (*dnaD*), DegV family protein (*degV*), TIGR00282 family metallophosphoesterase, Preprotein translocase SecY (*secY*), and RluA family pseudo uridine synthase (*rluA*)] were able to distinguish the strains of AY (16SrI) group of phytoplasmas into three different Operational Taxonomic Units (OTUs). This time, the authors deduced the five marker genes with high densities of informative genome sites and relatively high substitution rates. These selected genes were known not to deviate compared to other shared single-copy genes reported earlier. They were chosen based on their unique position in the distinct and separated genome regions depicting unlikely homologous recombination between the same OTUs (Cho et al., 2020).

The ICSB-Mollicutes subcommittee encouraged using multiple genetic markers, minimally 16S rRNA gene, and Elongation Factor-Tu (*EF-Tu*) to circumscribe the provisional taxa to which strains should be assigned (Firrao and Brown, 2013). Among all phytoplasma species published so far, the multi-locus sequence data of reference strain, in addition to 16S rRNA sequences, is available only for twelve species. Those are ‘*Ca.* P. asteris,’ ‘*Ca.* P. ulmi,’ ‘*Ca.* P. rubi,’ ‘*Ca.* P. solani,’ ‘*Ca.* P. balanitae,’ ‘*Ca.* P. meliae,’ ‘*Ca.* P. cirsii,’ ‘*Ca.* P. stylosanthis,’ ‘*Ca.* P. cynodontis,’ ‘*Ca.* P. sacchari,’ ‘*Ca*. P. tritici’ and ‘*Ca*. P. dypsidis’ where sequences of either *secY*, *tuf*, or *rp* genes are available (Table 1). Establishing a formal MLST system requires the availability of gene sequences of the reference strains. The phytoplasma multi-locus sequence database is currently less inclusive of the number of sequences available for phytoplasma reference strains of various phylogenetic groups or species. This also includes the related metadata of insect vectors or plant hosts and geographic locations. The availability and reproducibility of PCR primer pairs for proposed marker genes for each group of phytoplasmas species proved to be a primary hindrance in making the database comprehensive.

## The OGRI values: Key to unlocking phytoplasma taxonomy

The main objective of microbial taxonomy is to construct a comprehensive system that will encompass all three components: classification, nomenclature, and identification. In this context, genomics has become a promising methodology as it provides a stable, reproducible, and highly informative means to infer phylogenetic relationships among prokaryotes (Danet et al., 2011; Firrao et al., 2013; Chun and Rainey, 2014; Cho et al., 2020). This led to the construction of a dedicated curated databases, websites, and tools to aid the user-friendly taxonomic delineation of the prokaryotic strain under study (Yoon et al., 2017; Chaumeil et al., 2020; Parks et al., 2020, 2022; Parte et al., 2020). These recently employed methods recruit the sequence-derived numerical data to measure the distances among the prokaryotes, known as Overall Genome Relatedness Indices (OGRI) values (Kim et al., 2014; Barco et al., 2020).

The OGRI values are dDDH (digital DNA-DNA hybridization), G+C content, and ANI (Average Nucleotide Identity) values. The ANI values denote the percent identity inferred by the homologous regions shared between prokaryotic genomes and classify bacterial genomes (Jain et al., 2018). The Digital DDH (dDDH) ensures the highest consistency regarding the species-delimitation approach currently dominating microbial taxonomy. The DDH has been a gold standard in prokaryotic taxonomy that indirectly measures the degree of genetic similarity between two genomes (Chun et al., 2018). The minimal standards for using genome data for the taxonomy of prokaryotes are widely accepted that bacterial strains of the same species have ANI values >95–96% and DDH values >70% (Chun et al., 2018). The G+C content has been historically valued for taxonomic descriptions of species and genera (Meier-Kolthoff et al., 2014). A direct computation of G+C content with high accuracy is easily achieved from genome sequenced with considerable depth. The significance of accuracy achieved by this computation method over the drawbacks of using conventional methods (Meier-Kolthoff et al., 2014). These methods have helped delineate phytoplasma phylogenetic position with significant confidence compared to 16S rRNA gene phylogeny alone (Kirdat et al., 2021; Zhao et al., 2021). An analysis of 51 phytoplasma genomes revealed that some strains within the same clade in the 16S rRNA gene phylogenetic tree exhibited pairwise ANI values less than 95% and dDDH values less than 70% (unpublished data). This suggests that these phytoplasma strains could potentially be classified as new species, subject to the fulfillment of other necessary criteria for species designation.

A study consisting of 14 phytoplasma genomes was conducted to check the efficacy of OGRI values and phylogeny based on genomes (Firrao et al., 2013). For this analysis MLSA of 107 orthologous genes, Average Nucleotide Index (ANIm), tetranucleotide signature frequency correlation index (TETRA), and consensus networks were used. The authors found that the strains belonging to the same clade shared high ANI values confirming high relatedness at the genomic level, and relatively lower ANI values were calculated between strains belonging to two different subgroups of 16SrI. The TETRA values >990 would support phytoplasma species boundary based on the ANI range 95–96%; however, the TETRA index calculation can be inaccurate when comparing drafts based on different sequencing techniques (Firrao et al., 2013). In another attempt, 11 AY phytoplasma genomes were assigned to define the putative species boundaries and genomic divergence (Cho et al., 2020). The study proposed dividing the 16SrI group into three species-level OTUs based on whole-genome ANI analysis, homologous gene sequence-based analysis, PCoA analysis, and phylogenetic tree based on 303 single-copy-genes shared by selected 11 genomes. The study showed that the same OTU species have >97% ANI values, and species comparison between OTUs have <94% ANI values. The authors proposed the MLSA markers deduced from 11 analyzed AY genomes that could facilitate future genetic characterization of Aster Yellows phytoplasmas (AY) or 16SrI group (Cho et al., 2020). Homologous gene clusters showing genome divergence can further provide practical means to classify phytoplasma provided the availability of complete or near-complete genomes with completeness >90%.

The genome phylogeny and OGRI values were used to describe a new phytoplasma species, ‘*Ca*. P. sacchari,’ associated with Sugarcane Grassy Shoot (SCGS) disease (Kirdat et al., 2021). The phylogenetic position based on 16S rRNA and other SCGS phytoplasma genes was confusing due to distant branching from two species, ‘*Ca.* P. cynodontis’ and ‘*Ca.* P. oryzae.’ Many Rice Yellow Dwarf (RYD) group strains associated with Sugarcane Grassy Shoot (SCGS) disease of sugarcane were assigned to ‘*Ca.* P. oryzae’ erroneously despite their close phylogenetic association with ‘*Ca.* P. cynodontis’. An approach to constructing a hybrid assembly followed by metagenomic binning was used to obtain the SCGS phytoplasma genome (Kirdat et al., 2020). Using 13 near-complete phytoplasma genomes, the authors constructed the whole-genome phylogenetic tree using BPGA (Chaudhari et al., 2016) and UBCG (Na et al., 2018) tools to clear the confusion and to obtain a reliable and highly informative phylogenetic delineation using a whole-genome sequence of SCGS and related strains, OGRI values, and housekeeping genes. The comparative G+C content was directly calculated from the obtained draft genome assemblies and the dDDH values were calculated using Genome-to- Genome Distance Calculator (GGDC) webserver (http://ggdc.gbdp.org) (Auch et al., 2010). This approach provided a better phylogenetic position for sugarcane grassy shoot phytoplasma and its proposal as a novel taxon.

## Phytoplasma nomenclatural revisions according to ICNP guidelines

The phytoplasma species names are generally derived from their plant or insect host or a geographic location where the strain is endemic. The taxonomic notes and appendix 11 of the International Code of Nomenclature of Prokaryotes (ICNP) codes illustrate the guidelines to describe the ‘*Candidatus’* taxa (Murray and Schleifer, 1994; Murray and Stackebrandt, 1995; Garrity et al., 2015). Although the names proposed for ‘*Candidatus’* taxa do not come under ICNP rules, Oren (2017) proposed a nomenclature review for ‘*Candidatus’* taxa published in IJSEM and elsewhere (Oren, 2017). He evaluated more than 400 names, including provisional species names of *‘Candidatus* Phytoplasma,’ and suggested changes in the names in line with ICNP rules. He suggested nomenclature changes in three phytoplasma species ‘*Ca.* P. aurantifolia,’ ‘*Ca.* P. australasia,’ and ‘*Ca.* P. sudamericanum’ as ‘*Ca.* P. citri,’ ‘*Ca.* P. australasiaticum’ and ‘*Ca.* P. australamericanum’ or ‘*Ca.* P. meridianamericanum,’ respectively. ‘*Ca.* P. citri’ because the bacterium is found in the host plant, *Citrus aurantifolia*; the proposed name is, therefore, more appropriate. The name ‘aurantifolia’ means orange leaves, which cannot be a diagnostic characteristic of associated phytoplasma strain. ‘*Ca.* P. australasiaticum’ because ‘australasia’ is a noun, not an adjective. ‘*Ca*. P. australamericanum’ or ‘*Ca*. P meridianamericanum’ is because the suggested names are in Latin where ‘sudamericanum’ is a derived French word; this change is recommended according to the rule and recommendation 6 of ICNP (Garrity et al., 2015; Oren, 2017).

Additionally, in February 2021, the International Committee on Systematics of Prokaryotes (ICSP), who oversees the development and revision of the ICNP, proposed renaming the phylum *Tenericutes* to *Mycoplasmatota* (My.co.plas.ma.to’ta. N.L. neut. n. *Mycoplasma*, type genus of the phylum; -ota, ending to denote a phylum; N.L. pl. neut. n. *Mycoplasmatota*, the Mycoplasma phylum) to be included in the revised version of the ICNP (Oren and Garrity, 2021). The revised classification of phytoplasma reads: Kingdom, Bacteria; Phylum, *Mycoplasmatota*; class, *Mollicutes*; genus, *‘Candidatus* Phytoplasma’ (Parte et al., 2020).

## Toward a holistic approach in phytoplasma taxonomy

Recently, a ‘revision’ of phytoplasma species ‘guidelines’ were published with the recommendation to use ANI values and an update on increased cut-off value for 16S rRNA gene for the delineation of phytoplasma strains at the species level (Bertaccini et al., 2022). The article followed with detailed commentary on the proposed guidelines and emphasized the utility of RFLP-based grouping of phytoplasma strains (Wei and Zhao, 2022). Both these reviews covered the known aspects of phytoplasma taxonomy; highlighted some of the critical issues, however, they did not cover the matters related to validating published phytoplasma species and other vital elements of phytoplasma taxonomy discussed earlier in this review. This includes validation of ‘*Ca*. P. wodyetiae’ needs to be relooked for possible chimeric reference sequences. The confusion continues with a species status of ‘*Ca*. P. australasia’ based on the criteria suggested by the IRPCM 2004 and multiple studies published later. These articles did not shed light on the fate of orphan species where only one gene information is available, and no ‘-related strains’ were reported for those species. The ‘revised guidelines’ article retained these species with their reference strains. Additionally, the new species, ‘*Ca*. P. cocostanzaniae’ was proposed, represented by 16S rRNA gene sequence alone and no other data related to plant host or host range, insect vector(s), and other criteria laid by IRPCM, 2004 or later (Bertaccini et al., 2022). The taxonomical description of cultivable bacteria mandatorily demands the polyphasic characterization of the strain, including its physiological, biochemical, and genome characterization (Sneath, 1992). Therefore, the cut-off values for 16S rRNA gene percent similarity with existing species should be considered only a preliminary marker to delineate the phytoplasma strain to species level. The information gathered from the whole genome (OGRI values) and the endemic nature of its plant and insect host must be considered holistically to propose a new species of phytoplasma. It is detrimental to consider the fulfillment of ‘any one’ character as laid down by the IRPCM, 2004 or ‘revised guidelines’ or ‘suggested clarifications’ as a characteristic of phytoplasma species considering the challenges posed by the published species with limited data (Firrao, 2004; Bertaccini et al., 2022; Wei and Zhao, 2022).

**FIGURE 2:**
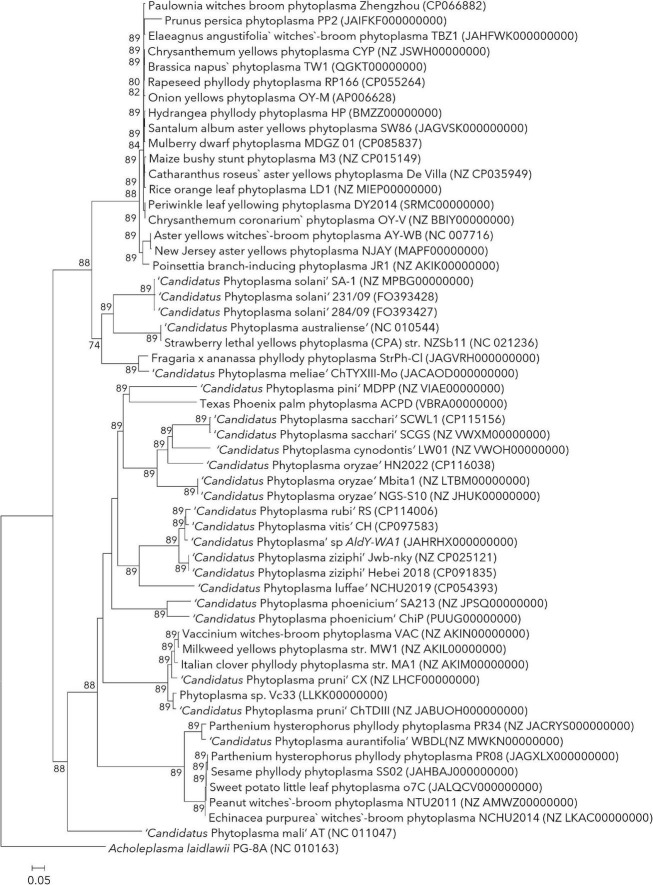
The pan-genome phylogenetic tree of phytoplasma based on orthologous gene sequences computed using the UBCG tool (Na et al., 2018). The length of UBCG concatenated alignment was 86,460 containing 89 marker genes identified using HMMER (Potter et al., 2018) and predicted using Prodigal (Hyatt et al., 2010) search. Figures at branch points are bootstrap values (>50). The genome of *Acholeplasma laidlawii* PG-8A (NC_010163) was used as an outgroup. Bar indicates the number of substitutions per site.

The situation of orphan species with limited information can be addressed by assigning neo reference strains whose genome information can be used for the comparative OGRI values. In a conventional bacterial taxonomy, the arrangement of a neo type strain is accepted by international agreement to replace a type strain that is no longer in existence to serve as the type strain (Gibbons, 1974; Tindall and Garrity, 2008; Oren and Garrity, 2020). Along similar lines, neo reference strains can be assigned to replace the published phytoplasma reference strains where OGRI and ecological information of proposed neo reference strains is available. The proposed neo reference strain must possess similar characteristics, if not identical, to that published reference strain with related information available freely to other researchers. The ‘revised guidelines’ are inconsistent with the terminology used for neo-reference strains and have used the terms ‘complementary’ and ‘alternative’ reference strains which may lead to further confusion. Also, it has assigned the strain 305/13 (KP019340) as a ‘complementary’ ‘additional’ strain along with published reference strain BGWL (AJ550984), despite the availability of the genome sequence of strain LW01 (VWOH00000000), which was proposed as neo reference strain (Kirdat et al., 2020, 2021). The complete genome sequence of OY-M (AP006628.2) can be used as a neo reference strain to replace the strain MIAY, a reference strain of ‘*Ca*. P. asteris’ (Lee et al., 2004a; Oshima et al., 2004). Similarly, the complete genome sequences of two strains (strain NCHU2014, CP040925; and strain PR08, CP097207) are available, and anyone can serve as a neo reference strain for ‘*Ca.* P. australasiaticum’ (formerly ‘*Ca*. P. australasia’). The strain SA213 (JPSQ0100000) can serve as a neo reference strain for ‘*Ca*. P. phoenicium’ replacing the published strain A4 (AF515636). The complete genome sequences of two strains, 284/09 and 231/09 (FO393427 and FO393428), can be assigned neo reference strain for ‘*Ca*. P. solani.’ This list can be extended to further with an analysis of available genome sequences for published species with limited OGRI information according to their phylogenetic position with mutual agreement at IRPCM meetings (Figure 2).

Currently, ‘*Candidatus’* species neither have standing in nomenclature nor have the protection of priority granted by the ‘Code.’ That means the current name need not be retained if the subsequent species is cultured onto synthetic media *in vitro*. Thus, ‘*Candidatus’* species cannot be published ‘validly’ or ‘officially.’ Also, the concept of ‘-related strain’ is well established in conventional bacterial taxonomy; the proposed concept of ‘member strains’ will add further confusion in terming the phytoplasma strains (Bertaccini et al., 2022; Wei and Zhao, 2022). None of the articles acknowledged the proposed changes in the names of ‘*Ca.* P. aurantifolia,’ ‘*Ca.* P. australasia,’ and ‘*Ca.* P. sudamericanum’ (Oren, 2017).

Phytoplasmas share a high variation in genome size, which re-emphasizes using a core gene set over a set of genes proposed for an MLSA (Chun et al., 2018; Cho et al., 2020; Kirdat et al., 2021). This approach avoids the complications caused due to loss or gain of genes induced by horizontal gene transfers, overestimating shared genomic regions due to varied genome sizes. The ‘revised guidelines’ article suggested using six marker genes (*tufB*, *secA, secY, rpIV-rpsC*, *groEL*, and 16S rRNA) to characterize phytoplasma strains; however, the basis of selection and cut-off similarity values of these genes are lacking (Bertaccini et al., 2022). These marker genes should be PCR amplifiable across the phytoplasma species, short enough but highly informative and robust against recombination. They should be distributed all over the genome so that horizontal gene transfer and genome rearrangement should have minimal effect on them (Cho et al., 2020). Attempts need to be directed toward obtaining whole genome sequences of phytoplasmas exhibiting large diversity, which should be utilized to devise a comprehensive taxonomic system by extracting the core gene set. Currently, more than 55 genome sequences representing less than 20% of species with published names are available (Figure 2). Out of those, twenty genomes represented by four taxonomic groups, only have been fully sequenced (Bai et al., 2006; Kube et al., 2008; Tran-Nguyen et al., 2008; Andersen et al., 2013; Wang et al., 2018; Cho et al., 2020; Kirdat et al., 2021).

One of the crucial criteria followed in conventional bacterial taxonomy is to make the biological resource, in the form of biological material, available for polyphasic comparative characterizations (Sneath, 1992). Several phytoplasma strains with provisional species status have been published with limited sequence information, mostly restricted to the 16S rRNA gene alone or a couple of housekeeping genes. The IRPCM, 2004 recommended making the biological resource material available, especially for reference strains- however, due to the Nagoya Protocol on Access and Benefit-sharing, the exchange of biological material is practically impossible or highly cumbersome (Greiber et al., 2012; Morgera et al., 2014; Smith et al., 2017). Most genome sequences deposited in the GenBank database are not backed with Sequence Archive Reads (SRA) data (the SRA stores raw sequencing data and alignment information to enhance reproducibility and facilitate discoveries through data analysis). This situation is detrimental and limits future taxonomic studies (Wei and Zhao, 2022). It is recommended that the resource material of reference strain be available freely to researchers in the form of the genome sequences.

## The path ahead: Charting the future

The phytoplasma taxonomy has undergone many transitions since its discovery and has witnessed different nomenclature and classification systems, and it should stabilize with the acceptance of binomial nomenclature. The scientific community is putting efforts into improving the taxonomy issues of ‘*Candidatus’* bacteria by proposing two potential paths that will allow these organisms to be described based on genome data and predicted functional characters. Many researchers have proposed a path that has been discussed for a long time to allow DNA sequence as type material (Murray and Schleifer, 1994; Murray and Stackebrandt, 1995; Sneath, 2005; Konstantinidis et al., 2017; Jain et al., 2018; Oren and Garrity, 2018). This proposal suggests amendments in ICNP rules within its framework and tries to establish a common nomenclature system to be used by both cultivated and uncultivated taxa. This proposal also suggests that the whole genome is not needed for each species; rather, multi-locus sequence typing can be sufficient to identify a species unambiguously (Whitman, 2016; Whitman et al., 2019). The alternate path toward comprehensive nomenclature is to create a parallel nomenclature system for uncultivated taxa, probably named ‘The Uncultivated Code’ or ‘SeqCode’ (Murray et al., 2020; Hedlund et al., 2022; Palmer et al., 2022; Whitman et al., 2022). The taxon names will be associated with high-quality descriptions, including genome data meeting a set of minimal standards, metadata about the habitat, bioinformatics-based functional predictions, and microscopic images of organisms confirmed by *in situ* hybridization. The current proposal intends to merge this parallel code in the ICNP later in the future. Both paths are destined to stabilize the nomenclature of uncultivated taxa and ascertain priority. These developments need to be adapted by the phytoplasma research community for refined and stable phytoplasma taxonomy with due discussion and debate.

The broader acceptance and consensus of all phytoplasmologists on adopting comprehensive methods in classifying the phytoplasma strains is required where IRPCM can play a pivotal role.

## Author contributions

KK and BT carried out the phylogenetic analysis and data compilation. KK wrote the first draft. SS revised and edited the first draft. AY conceptualized, revised, and finalized the manuscript. All authors, read, and agreed to the final version of the manuscript.
